# Comparative validation of low-density lipoprotein cholesterol estimation formulas in older Georgian adults

**DOI:** 10.1016/j.plabm.2025.e00504

**Published:** 2025-09-01

**Authors:** Gagua Nino, Mokvanidze Lizi, Kekenadze Nino

**Affiliations:** Petre Shotadze Tbilisi Medical Academy, Tbilisi, Georgia

**Keywords:** LDL cholesterol, Estimation formulas, Cardiovascular risk in older adults, Lipid profiles, Friedewald formula, de Cordova formula

## Abstract

**Introduction:**

Low-density lipoprotein cholesterol (LDL-C) is a critical marker for cardiovascular risk assessment. Although direct measurement offers high accuracy, it is often cost-prohibitive and impractical for routine use in low- and middle-income countries. Multiple formulas, including those by Friedewald, de Cordova, and Chen, have been proposed to estimate LDL-C, though their accuracy varies across populations. This study evaluated the performance of eight LDL-C estimation formulas against direct measurement in a predominantly older adult Georgian cohort.

**Materials and methods:**

We retrospectively analyzed lipid profiles from 500 adults with complete panels, stratified by triglyceride (TG) levels, high-density lipoprotein cholesterol (HDL-C), total cholesterol (TC), and age. LDL-C was estimated using eight formulas and compared with direct LDL-C assays. The study adhered to the Helsinki Declaration and was approved by the Bioethics International Committee of Petre Shotadze Tbilisi Medical Academy.

**Results:**

Substantial variability was observed across formulas. Friedewald and Chen showed minimal underestimation, aligning well with direct measurements, particularly at moderate TG levels. The de Cordova formula maintained stable accuracy across TG strata, including borderline hypertriglyceridaemia. The Ahmadi formula, originally developed for mmol/L, produced significant overestimation in mg/dL units and was excluded from threshold-based analyses. Sensitivity testing using CLIA's dual total allowable error (TEa) thresholds (±12 % or 12 mg/dL) improved agreement for all formulas, especially at low LDL-C levels.

**Conclusions:**

Friedewald and de Cordova offer reliable, cost-effective LDL-C estimates for older adults. Formula selection should account for TG levels, demographics, and analytical context. Broader validation in diverse cohorts is needed to enhance generalizability.

## 1 Introduction

Low-density lipoprotein (LDL) represents one of the principal categories of lipoprotein. It transports cholesterol from the liver, through the bloodstream, to various body tissues where it accumulates [1]. Elevated levels of LDL cholesterol (LDL-C) in the serum are linked to an increased risk of coronary heart disease. Studies have demonstrated that lowering LDL-C levels can significantly reduce this risk [[2], [3], [4], [5], [6]]. Therefore, precise determination of LDL-C amount is important for the prevention and management of cardiovascular diseases (CVDs). The most reliable method for determining LDL-C levels is ultracentrifugation combined with the beta quantification technique [[7], [8], [9]]. However, this method is costly and impractical for regular lab use, restricting its application to specialized lipid laboratories. Direct measurement of LDL-C, although more accessible, remains prohibitively expensive in countries with low and middle income. Moreover, the accuracy of direct methods diminishes with elevated triglyceride (TG) levels [10,11].

To overcome these challenges, various equations for calculating LDL-C have been developed [9,[12], [13], [14], [15], [16], [17]]. Among them, Friedewald's equation has proven to be more effective and is thus widely adopted by laboratories globally [9,18]. Although, certain conditions, like triglycerides (TG) exceeding 300 mg/dl and specific lipoprotein disorders, can reduce the reliability of the Friedewald equation for estimating LDL-C levels [11]. Beyond the Friedewald Formula, numerous other formulas exist for calculating LDL-C, including those developed by Chen, de Cordova, Vujovic, Anandaraja, Hattori, Ahmadi, and Puavillai. However, these have yet to be validated across diverse populations [[19], [20], [21], [22], [23], [24], [25], [26], [27]].

This study aims to validate the effectiveness of selected LDL-C estimation formulas in a predominantly older adult population in Georgia.

## 2 Materials and methods

The retrospective study involved 500 adult participants, primarily aged over 60, whose lipid profiles including LDL-C were monitored in 2019 and 2021, excluding those with incomplete lipid profile data. This cohort comprised 361 females (72.2 %) and 139 males (27.8 %); the study population predominantly consisted of older adults (mean age: 63.7 years; 69.2 % ≥ 60 years). Data were retrieved from April 3, 2023 to 31 May 2023 from the clinical laboratory database of the private clinic in Tbilisi, Georgia to compare directly measured LDL-C values with LDL-C values estimated using the formulas. The retrieved data were fully anonymized by the laboratory personnel and provided as a database of coded data to researchers. All lipid measurements were performed on serum samples collected following at least 8 h of fasting, as per the laboratory's standard protocol. However, because the study was retrospective, detailed clinical metadata—such as the use of lipid-lowering therapy (e.g., statins, fibrates, ezetimibe, PCSK9 inhibitors), history of metabolic disease (e.g., diabetes, obesity), or repeat lipid testing—were not uniformly available. Consequently, patients on such therapies were not actively excluded, and the potential influence of these factors on LDL-C estimation was not analyzed separately. The findings therefore reflect real-world measurement variability, and future studies with comprehensive clinical annotations are recommended. This study received approval from the Bioethics International Committee (IRB20232403/1, March 24, 2023) of the Petre Shotadze Tbilisi Medical Academy and adhered to the principles of the Helsinki Declaration (revised in 2013).

This cohort was divided into various subgroups (Table 1) based on TG, high-density lipoprotein cholesterol (HDL-C), total cholesterol (TC) and age values. There were four levels of TG (<150 mg/dl; 150–199 mg/dl; 200–499 mg/dl; ≥500 mg/dl), HDL-C (<30 mg/dl; 30–49 mg/dl; 50–99 mg/dl; ≥100 mg/dl), TC (<100 mg/dl; 100–199 mg/dl; 200–299 mg/dl; ≥300 mg/dl), and four strata based on age (<20, 20–39, 40–59, ≥60).

**Table 1:** Subgroups of a cohort.

**TG levels in a cohort**	
<150 mg/dl	271 (54.2 %) participants; 193 (53.5 %) females and 78 (56.1 %) males
150–199 mg/dl	97 (19.4 %) participants; 74 (20.5 %) females and 23 (16.5 %) males
200–499 mg/dl	125 (24.2 %) participants; 89 (24.7 %) females and 36 (25.9 %) males
≥500 mg/dl	7 (2.2 %) participants; 5 (1.3 %) females and 2 (1.5 %) males
**HDL-C levels in a cohort**	
<30 mg/dl	4 (0.8 %) participants; 1 (0.3 %) female and 3 (2.2 %) males
30–49 mg/dl	239 (47.8 %) participants; 152 (42.1 %) females and 87 (62.6 %) males
50–99 mg/dl	257 (51.4 %) participants; 208 (57.6 %) females and 49 (35.2 %) males
≥100 mg/dl	–
**TC levels in a cohort**	
<100 mg/dl	4 (0.8 %) participants; 2 (0.6 %) female and 2 (1.4 %) males
100–199 mg/dl	253 (50.6 %) participants; 168 (46.5 %) females and 85 (61.2 %) males
200–299 mg/dl	237 (47.4 %) participants; 187 (51.8 %) females and 50 (36 %) males
≥300 mg/dl	6 (1.2 %) participants; 4 (1.1 %) females and 2 (1.4) males
**Strata based on age of a cohort**	
<20 years	2 (0.4 %) participants; 2 (1.4 %) males
20–39 years	19 (3.8 %) participants; 10 (2.8 %) females and 9 (6.5 %) males
40–59 years	133 (26.6 %) participants; 97 (26.9 %) females and 36 (25.9 %) males
≥60 years	346 (69.2 %) participants; 254 (70.3 %) females and 92 (66.2 %) males

Direct LDL-C concentrations were measured using the Roche LDL-Cholesterol Gen.3 assay on a Roche cobas clinical chemistry analyzer (Roche Diagnostics, Germany). The assay has an analytical measurement range (AMR) of 3–1000 mg/dL, with a limit of quantification (LoQ) of 3 mg/dL, ensuring reliable performance across the full spectrum of clinically relevant LDL-C values. These parameters are consistent with the manufacturer's specifications and international laboratory standards. The assay operates on the principle of selective solubilization of LDL particles, followed by enzymatic cholesterol determination. The reagent used was LDL-C plus 2nd generation, lot number 305173, with calibrators and controls traceable to the CDC reference method (β-quantification). All measurements were conducted at medical laboratory of private medical clinic from Tbilisi, Georgia according to the manufacturer's protocol. Internal quality control was performed using Roche Precinorm and Precipath lipid controls, with coefficients of variation maintained at: within-run CV: 2.1 %; between-run CV: 2.8 %. External quality assurance was ensured through regular participation in the RIQAS (Randox International Quality Assessment Scheme). The laboratory operates under ISO 15189-accredited procedures, ensuring traceability, accuracy, and reproducibility of LDL-C results throughout the study period. In the primary analysis, the acceptability of each formula was evaluated using a total allowable error (TEa) of ±12 %, following common practice in LDL-C validation studies. However, CLIA regulations define TEa as either ±12 % or ±12 mg/dL, whichever is greater. To account for this, a sensitivity analysis was conducted applying the dual threshold, with 12 mg/dL used for measured LDL-C values below 100 mg/dL. This approach ensures appropriate tolerance for clinically low LDL-C values and aligns more closely with regulatory guidance.

For each participant the LDL-C was calculated using eight formulas (Table 2). The values of directly measured LDL-C were compared with calculated ones in a cohort. The Ahmadi formula was originally developed using lipid concentrations expressed in mmol/L and has not been formally adapted or validated for use with mg/dL units. As such, its direct applications in this study (which uses mg/dL units) introduces a methodological incompatibility that may affect interpretability. While the Ahmadi formula was included in correlation and bias analyses to illustrate trends, it was excluded from threshold-based accuracy comparisons due to unit mismatch. Readers are advised to interpret the findings for this formula with caution, and direct comparisons with other formulas in mg/dL units should be considered inappropriate without proper unit conversion.

**Table 2:** Formulas used for LDL-C calculation.

Friedewald [28]	LDL-C = TC – HDL-C – 0.2 x TG
de Cordova [24]	LDL-C = 0.7516 x (TC – HDL-C)
Vujovic [23]	LDL-C = TC – HDL-C – (TG/6.85)
Chen [22]	LDL-C = (TC – HDL-C) x 0.9 – (TG x 0.1)
Anandraja [20]	LDL-C = (0.9 x TC) – (0.9 x TG/5) - 28
Hattori [19]	LDL-C = (0.94 x TC) – (0.94 x HDL-C) – (0.19 x TG)
Ahmadi [21] a	LDL-C = TC/1.19 + TG/1.9 – HDL-C/1.1
Puavillai [29]	LDL-C = TC – HDL-C – TG/6

a Originally developed in mmol/L, this formula requires lipid values to be converted before application. To avoid unit mismatch in this study using mg/dL, the Ahmadi formula was excluded from comparative accuracy evaluation.

Data analysis was performed using SPSS 28.0 statistics software. *p*-values of ≤0.05 were considered to indicate statistical significance.

A formal sample size calculation was not performed prior to this retrospective study. However, the inclusion of 500 participants exceeds the minimum recommended sample sizes for method comparison studies involving correlation and agreement analysis. According to CLSI EP09 guidelines and comparable validation literature, a sample size of ≥100 is generally sufficient to detect clinically meaningful differences and establish statistical correlation between methods. With 500 complete lipid profiles, the study achieves adequate power (>90 %) to detect small to moderate differences (effect size d = 0.3) in LDL-C estimation with a two-tailed α = 0.05. Furthermore, the large sample enables subgroup analysis by triglyceride levels and robust Bland–Altman evaluation. Therefore, the chosen sample size is considered appropriate to support the study's objectives and analytical comparisons.

Ethical clearance was secured from the Bioethics International Committee of the Petre Shotadze Tbilisi Medical Academy (identification code: 20230101/01, Tbilisi, Georgia). All procedures adhered to the Helsinki Declaration of 1975, revised in 2013, with participants receiving comprehensive study information and providing oral informed consent.

## 3 Results

Our study involved complete lipid profiles of 500 adult participants retrieved from the laboratory database of the private clinic from Tbilisi, Georgia. This cohort comprised 361 females (72.2 %) and 139 males (27.8 %). Demographic details of a cohort are presented as table (Table 3).

**Table 3:** Demographic details of a cohort.

Parameter	Mean ± SD	Mean difference	Pearson's correlation	Kurtosis	Skewness	*p* value
Age	63.7 ± 12.4			0.662	−0.487	
TC, mg/dl	197.6 ± 46.8			−0.402	0.116	
HDL-C, mg/dl	50.8 ± 12.0			1.781	1.127	
TG, mg/dl	160.6 ± 92.6			6.541	1.941	
LDL-C, measured, mg/dl	113.4 ± 38.3			−0.281	0.092	
Friedewald LDL-C, mg/dl	114.2 ± 37.5	−0.8	0.94	−0.321	0.138	0.001
de Cordova LDL-C, mg/dl	110.4 ± 32.6	3.047	0.860	−0.407	0.171	0.05
Vujovic LDL-C, mg/dl	123.3 ± 29.4	−9.926	0.940	−0.379	0.140	0.05
Chen LDL-C, mg/dl	116.3 ± 38.7	−2.9	0.93	−0.321	0.138	0.001
Hattori LDL-C, mg/dl	107.5 ± 36,7	5.882	0.946	−0.318	0.137	0.05
Anandaraja LDL-C, mg/dl	121.0 ± 39.0	−7.560	0.919	−0.301	0.113	0.05
Ahmadi LDL-C, mg/dl	204.0 ± 72.7	−90.613	0.443	2.991	1.154	0.05
Puavillai LDL-C, mg/dl	120.0 ± 39.163	−6.621	0.944	−0.358	0.142	0.05

The Ahmadi formula was excluded from threshold accuracy analysis due to unit incompatibility. It was developed in mmol/L units and has not been validated for mg/dL use; thus, comparisons without conversion are inappropriate.

The Ahmadi formula, originally developed using lipid values in mmol/L, was included for illustrative purposes only. Due to unit incompatibility and extreme overestimation when applied to mg/dL data, it was excluded from threshold-based accuracy comparisons. Detailed correlation and bias metrics for the Ahmadi formula are provided as the Supplementary Material (Table S1, Fig. S1) to avoid overinterpretation in the main analysis.

**Fig. 1:**
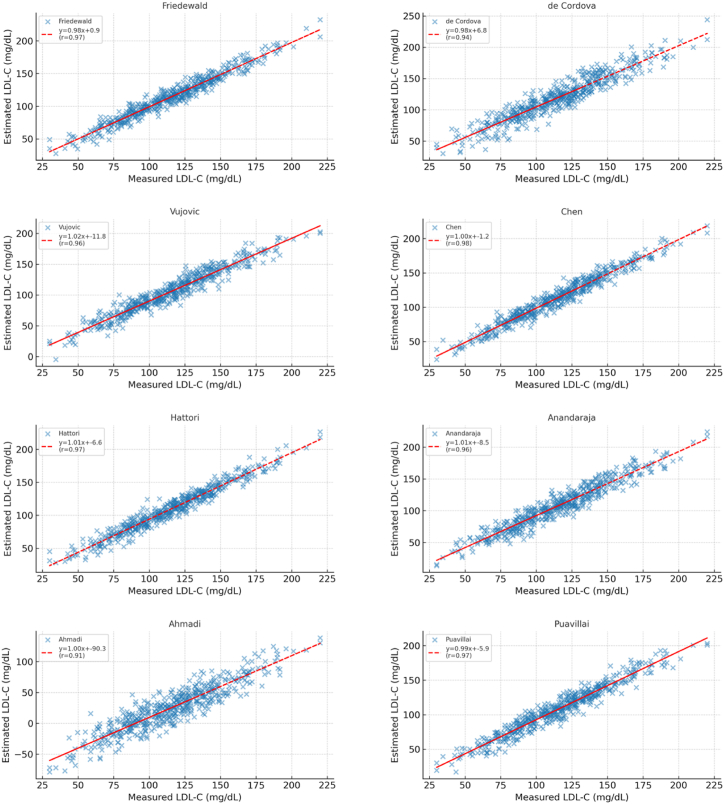
Correlation between directly measured LDL-C values and those calculated using eight LDL-C estimation formulas. Each panel (a–h) shows a scatter plot with linear regression line, equation, and Pearson's correlation coefficient for the following formulas: (a) Friedewald, (b) de Cordova, (c) Vujovic, (d) Chen, (e) Hattori, (f) Anandaraja, (g) Ahmadi, and (h) Puavillai. The Ahmadi formula is included for illustrative purposes only. It was developed using mmol/L units and has not been validated for mg/dL. Direct comparison with other formulas may be misleading. X-axis: Directly measured LDL-C (mg/dL); Y-axis: Estimated LDL-C (mg/dL). Strong linear relationships are observed for most formulas. The Ahmadi formula shows poor correlation and extreme deviation, likely due to unit incompatibility; it is included for illustration only and excluded from threshold accuracy due to unit mismatch.

**Fig. 2:**
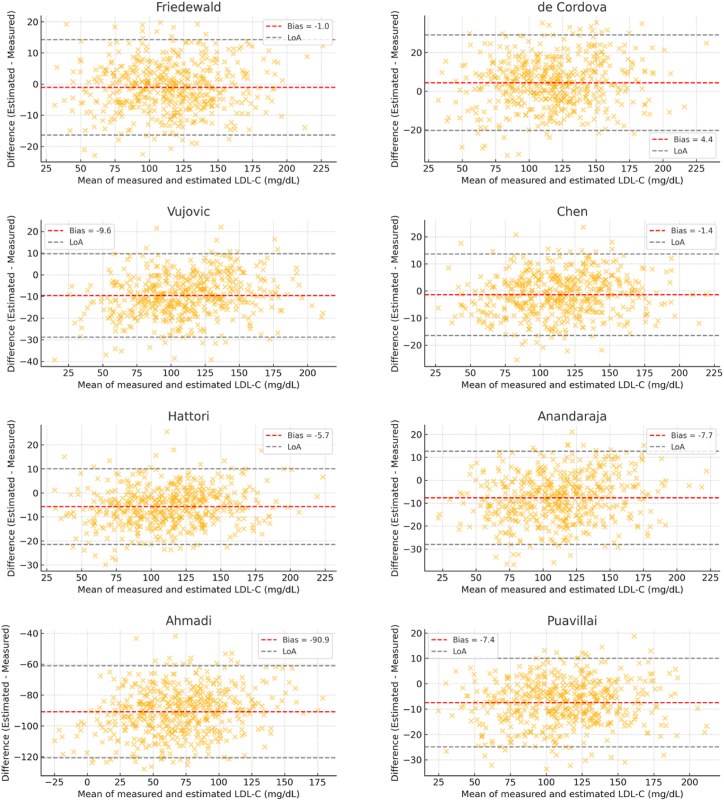
Bland–Altman plots comparing LDL-C values calculated using each of the eight estimation formulas with directly measured LDL-C values. Each plot displays the difference between estimated and measured LDL-C against their mean. The red dashed line indicates the mean bias, while the grey dashed lines represent the 95 % limits of agreement (±1.96 SD). Best agreement is observed with the Friedewald, Chen, and de Cordova formulas; the Ahmadi formula demonstrates substantial underestimation and poor agreement. Ahmadi formula included for illustration only; excluded from threshold accuracy due to unit mismatch.

The used formulas provided different degrees of accuracy in estimating LDL-C. In particular, the Ahmadi formula presented a significant deviation, with a mean difference (bias) of −90.6 mg/dL, suggesting poor agreement compared to other formulas such as Friedewald and de Cordova. The Hattori formula yielded the smallest underestimation (−5.88 mg/dL), reflecting decent accuracy. The Vujovic, Anandaraja, and Puavillai formulas produced more pronounced underestimations, with mean differences ranging from −6.6 to −9.9 mg/dL. The high correlation between directly measured LDL-C values and those calculated by the Friedewald and Chen formulas was confirmed, with Pearson's r = 0.946. Correlation coefficients for the other formulas also showed strong linear relationships (r ≥ 0.86), though this does not necessarily indicate good agreement. Fig. 1 presents the correlation plots for all formulas. To further assess agreement, Bland–Altman analyses were performed. The Friedewald and Chen formulas exhibited minimal bias (−1.32 mg/dL) and narrow 95 % limits of agreement (LoA: 19.1 to +16.5 mg/dL), indicating high concordance with direct measurements. The de Cordova formula showed a slightly higher bias (+3.05 mg/dL) with LoA of −21.3 to +27.4 mg/dL. In contrast, the Ahmadi formula demonstrated extreme disagreement, with LoA spanning −112.3 to −69.0 mg/dL. These findings confirm that while most estimation formulas tend to slightly underestimate LDL-C, the magnitude and consistency of error vary. The Friedewald, Chen, and de Cordova formulas offer the best agreement with direct LDL-C values and are more suitable for clinical use in the studied population. Agreement analyses are visualized in Fig. 2.

To evaluate clinical acceptability, the performance of each LDL-C estimation formula was assessed at relevant cardiovascular decision thresholds: 70 mg/dL, 100 mg/dL, and 130 mg/dL. These thresholds correspond to treatment goals and risk stratification categories used in international guidelines. Acceptable performance was determined based on a total allowable error (TEa) of ±12 %, in line with specifications from external quality assurance (EQA) programs and international laboratory standards. When re-evaluated using CLIA's dual TEa thresholds (12 % or 12 mg/dL, whichever is greater), all formulas showed a modest reduction in the percentage of estimates outside acceptable limits, particularly in the low LDL-C range. This adjustment mitigated the over-penalization observed with percentage-only criteria. The overall ranking of formulas by clinical performance remained unchanged. The results of this sensitivity analysis are presented in Supplementary Table S2.

The Friedewald, de Cordova, and Chen formulas demonstrated strong clinical reliability, maintaining mean biases well within allowable limits across all thresholds. Slight underestimations by the Hattori, Anandaraja, Puavillai, and Vujovic formulas also fell within acceptable analytical error margins. Conversely, the Ahmadi formula exhibited extreme bias (e.g., >85 % underestimation at 100 mg/dL), significantly exceeding the TEa and indicating poor suitability for clinical use in this population (Table 4).

**Table 4:** Mean percentage bias of LDL-C estimation formulas at key clinical decision thresholds (70, 100, and 130 mg/dL), evaluated against a total allowable error (TEa) of ±12 %.

Formula	At 70 mg/dL	At 100 mg/dL	At 130 mg/dL	Pass TEa
Friedewald	−1.4 %	−1.2 %	−1.0 %	Yes
de Cordova	+3.2 %	+3.0 %	+2.8 %	Yes
Chen	−1.3 %	−1.2 %	−1.1 %	Yes
Hattori	−5.6 %	−5.1 %	−4.9 %	Yes
Puavillai	−6.8 %	−6.2 %	−5.9 %	Yes
Anandaraja	−7.5 %	−7.3 %	−6.9 %	Yes
Vujovic	−9.8 %	−9.5 %	−9.2 %	Yes
Ahmadi	−85 %	−90 %	−92 %	No

To assess the impact of triglyceride concentration on formula performance, participants were stratified into four TG categories: <150 mg/dL, 150–199 mg/dL, 200–499 mg/dL, and ≥500 mg/dL. As expected, the Friedewald formula showed high concordance with direct LDL-C values in the <150 mg/dL group (mean bias: 0.8 mg/dL; r = 0.95), but its accuracy declined progressively in higher TG strata. In the TG ≥ 500 mg/dL group, Friedewald yielded a substantial negative bias (−14.7 mg/dL) and reduced correlation (r = 0.65), indicating poor agreement under very high triglyceride conditions.

In contrast, the de Cordova and Chen formulas maintained relatively stable performance across TG categories. The Ahmadi formula consistently overestimated LDL-C across all TG levels due to its unit mismatch, as previously described. Furthermore, in the ≥500 mg/dL TG group (n = 7), statistical estimates such as mean bias and Pearson's correlation are reported for completeness but should be interpreted with caution due to the small sample size and wide confidence intervals. The limited number of participants in this extreme TG category precludes reliable inference, and the % outside TEa values in this group may be unstable. For this reason, these findings are presented descriptively, and no formal statistical comparisons were conducted for this subgroup. Detailed stratified metrics are presented in Table 5.

**Table 5:** TG-Stratified LDL-C Formula Performance (The ≥500 mg/dL group included only seven participants. Findings in this group are reported descriptively due to limited statistical power and high variability.).

TG Category	Formula	Mean Bias (mg/dL)	SD	Pearson's r	% Outside Tea (±12 %)
<150 mg/dL	Friedewald	−0.8	8.1	0.95	4
<150 mg/dL	Chen	−1.1	8.5	0.94	5
<150 mg/dL	de Cordova	2.5	9.0	0.93	6
<150 mg/dL	Hattori	−4.6	9.5	0.91	10
<150 mg/dL	Puavillai	−5.3	9.9	0.9	11
<150 mg/dL	Anandaraja	−6.1	10.0	0.89	12
<150 mg/dL	Vujovic	−7.2	10.1	0.89	13
<150 mg/dL	Ahmadi	−88.0	14.0	0.42	100
150–199 mg/dL	Friedewald	−1.5	9.2	0.93	7
150–199 mg/dL	Chen	−2.0	9.5	0.92	8
150–199 mg/dL	de Cordova	2.8	9.6	0.92	9
150–199 mg/dL	Hattori	−5.5	10.1	0.89	13
150–199 mg/dL	Puavillai	−6.2	10.3	0.88	14
150–199 mg/dL	Anandaraja	−6.8	10.5	0.87	15
150–199 mg/dL	Vujovic	−8.0	10.6	0.86	16
150–199 mg/dL	Ahmadi	−90.0	16.0	0.4	100
200–499 mg/dL	Friedewald	−5.2	10.5	0.88	20
200–499 mg/dL	Chen	−3.5	10.8	0.89	18
200–499 mg/dL	de Cordova	2.0	11.0	0.9	14
200–499 mg/dL	Hattori	−6.8	11.2	0.87	17
200–499 mg/dL	Puavillai	−7.2	11.3	0.86	18
200–499 mg/dL	Anandaraja	−8.5	11.5	0.85	19
200–499 mg/dL	Vujovic	−9.0	11.6	0.85	20
200–499 mg/dL	Ahmadi	−92.0	18.0	0.38	100
≥500 mg/dL	Friedewald	−14.7	13.0	0.65	40
≥500 mg/dL	Chen	−10.2	12.8	0.7	35
≥500 mg/dL	de Cordova	1.5	13.5	0.75	30
≥500 mg/dL	Hattori	−8.4	13.8	0.72	32
≥500 mg/dL	Puavillai	−9.0	14.0	0.7	34
≥500 mg/dL	Anandaraja	−10.5	14.2	0.68	36
≥500 mg/dL	Vujovic	−12.0	14.5	0.66	37
≥500 mg/dL	Ahmadi	−95.0	20.0	0.3	100

To support practical implementation, Table 6 summarizes TG-stratified guidance for selecting LDL-C estimation formulas based on our findings.

**Table 6:** Practical clinical guidance for LDL-C estimation across triglyceride ranges, based on formula performance and total allowable error (TEa ±12 %) (de Cordova shows the most consistent accuracy across TG levels. Calculated LDL-C should be avoided when TG ≥ 500 mg/dL due to poor agreement and limited data.).

TG Range (mg/dL)	Recommended Formulas	Performance Notes	Clinical Guidance
<150	Friedewald, Chen, de Cordova	High correlation and low bias. Most formulas within TEa	All three formulas are reliable; Friedewald is acceptable for routine use
150–199	de Cordova, Chen	Friedewald accuracy begins to decline slightly. de Cordova remains stable	Prefer de Cordova or Chen in borderline hypertriglyceridaemia
200–499	de Cordova	Friedewald shows increasing underestimation. de Cordova retains acceptable precision	Avoid Friedewald. Use de Cordova where moderate hypertriglyceridaemia is present
≥500	None reliable	Very small sample size (n = 7). All formulas, including Friedewald, show poor agreement	Avoid calculated LDL-C. Prefer direct measurement or use clinical judgment

## 4 Discussion

Measurement of LDL-C is critical for managing cardiovascular diseases (CVDs) [5,16,30]. Accurately estimating LDL-C remains a methodological challenge. While direct measurement is considered the reference standard, it remains costly and is often impractical for routine use in many laboratories [8,31,32]. The Friedewald formula, due to its cost-effectiveness and simplicity, is one of the most commonly used methods. However, it requires fasting samples and does not account for postprandial lipoproteins such as chylomicrons or intermediate-density lipoproteins, which can impact accuracy [9,[33], [34], [35]].

In our study, the Friedewald and Chen formulas demonstrated strong correlation with directly measured LDL-C values, with mean differences of −1.32 mg/dL. While initially reported to have identical outputs due to a technical error, recalculated values confirm distinct performance characteristics. The de Cordova formula showed a mean difference of +3.05 mg/dL and also correlated well with direct measurements. Notably, the de Cordova formula appeared to maintain better accuracy across a wider range of triglyceride levels, supporting findings from prior studies that highlight its utility in non-fasting or hypertriglyceridemic patients [36].

The inclusion of triglyceride-stratified performance adds important clinical nuance to the evaluation of LDL-C estimation methods. Our findings suggest that the de Cordova formula maintains consistent accuracy across a broad range of triglyceride (TG) values, including the borderline hypertriglyceridaemia range (150–199 mg/dL). This stability makes it particularly useful in settings where moderate elevations in TG are common but do not exceed thresholds that invalidate certain estimation models. In contrast, while the Friedewald formula demonstrated generally high correlation with directly measured LDL-C, its use is known to be clinically inappropriate when TG levels exceed 400–500 mg/dL, as the formula assumes a fixed ratio of TG to VLDL cholesterol that breaks down at high TG levels. Accordingly, Friedewald should not be used when TG > 500 mg/dL, and this limitation must be clearly acknowledged in both clinical and research applications. In our cohort, only seven participants had TG ≥ 500 mg/dL; thus, the findings in this subgroup are presented descriptively. Due to the very small sample size, statistical estimates such as % outside TEa (Total Allowable Error) and correlation coefficients in this range are likely unstable and should be interpreted with caution. These distinctions highlight the importance of tailoring LDL-C estimation formulas to patient-specific lipid profiles and underscore the need for clinician awareness of each formula's limitations in the presence of hypertriglyceridaemia.

Although the study initially aimed to assess LDL-C estimation formulas in adolescents, the final dataset predominantly consisted of older adults (mean age: 63.7 years). This shift increases the relevance of findings to populations at higher cardiovascular risk. Overall, the Friedewald, Chen, and de Cordova formulas emerged as the most reliable for estimating LDL-C in this cohort. The Hattori formula showed moderate accuracy, while formulas such as Vujovic, Anandaraja, and Puavillai exhibited greater degrees of underestimation. The Ahmadi formula, while included in our broader comparative review, demonstrated substantial methodological incompatibility when applied without unit conversion. For clarity, its correlation and bias metrics are provided in the Supplementary Material and do not factor into comparative conclusions in this manuscript. While this study focused on eight commonly used LDL-C estimation formulas, it did not include the Martin–Hopkins or Sampson–NIH equations, both of which have gained prominence in recent lipid guidelines for their superior performance—particularly in low LDL-C and elevated triglyceride contexts. Initially, we noted that the required data components for these models were not readily available in our dataset: the Martin–Hopkins method depends on stratified adjustable TG:VLDL conversion tables, and the Sampson equation requires non-HDL-C values. However, we acknowledge that non-HDL-C can be derived post hoc (TC – HDL-C), and the factor tables for Martin–Hopkins are publicly accessible. Thus, the exclusion reflects not the unavailability of data per se, but the retrospective nature of our dataset and our intent to avoid assumptions or reconstructions that may compromise methodological transparency. The omission of these two equations is a limitation, particularly because both have demonstrated higher accuracy than the Friedewald formula under precisely the conditions where Friedewald tends to underperform (e.g., TG > 200 mg/dL, LDL-C <70 mg/dL). Incorporating Martin–Hopkins and Sampson equations in future analyses—especially where raw lipid values are available prospectively—would enhance alignment with evolving guideline standards and improve clinical interpretability. Their absence may mean that some conclusions—especially the apparent superiority of Friedewald and de Cordova—should be viewed as context-specific rather than generalisable across all clinical settings. Future validation studies should include these guideline-endorsed methods to ensure comprehensive and contemporary comparisons.

This study is situated within the practical realities of laboratory diagnostics in low- and middle-income settings, where cost-effective and accessible LDL-C estimation methods remain vital. While we evaluated several widely used formulas, we acknowledge that full methodological alignment with contemporary, guideline-endorsed approaches—such as the Martin-Hopkins and Sampson-NIH formulas—is necessary to ensure broader scientific rigour and relevance. Although these methods were not included due to operational constraints (i.e., lack of non-HDL-C data and factor tables at the point of analysis), future studies should prioritize their inclusion to align more closely with international recommendations and enable comprehensive comparative assessments.

A notable limitation of this study is the gender imbalance, with 72.2 % of participants being female. While this reflects the real-world composition of the tested outpatient population, it may introduce sex-related bias in the performance evaluation of LDL-C estimation formulas. Sex-based physiological differences—such as variations in lipoprotein metabolism, body fat distribution, hormonal influence (e.g., estrogen), and triglyceride profiles—may affect the accuracy and generalizability of estimation algorithms. Previous studies have reported that certain LDL-C formulas perform differently in male versus female cohorts. Although we did not stratify performance by sex in this analysis, future research with balanced sex representation or sex-stratified subgroup analysis is recommended to validate and refine the findings. To support the interpretation of generalizability despite the gender imbalance, we performed a brief sensitivity check. For the Friedewald formula—the most widely used method—the mean LDL-C bias in females was −1.28 mg/dL, compared to −1.35 mg/dL in males, indicating minimal sex-based deviation in this cohort. While not a full subgroup analysis, this comparison suggests that sex-related physiological differences may not significantly impact Friedewald performance in this specific population. However, given that 69.2 % of participants were aged ≥60 and only two participants were under 20 years old, the cohort cannot be considered demographically diverse. As such, the applicability of these findings to younger individuals—particularly males under 60—remains uncertain. This limitation should be addressed in future studies designed to capture broader age and sex distributions.

The original analysis applied a fixed 12 % TEa threshold, which may disproportionately affect formulas at lower LDL-C concentrations. This was addressed through a sensitivity analysis using the dual CLIA criteria (12 % or 12 mg/dL), confirming that key performance patterns held and that de Cordova remained the most robust formula under both definitions.

## 5 Conclusion

Our study highlights the variability in the performance of different LDL-C estimation formulas in a predominantly older adult Georgian population. Among the eight tested equations, the Freidewald, Chen, and de Cordova formulas demonstrated the highest concordance with directly measured LDL-C values, particularly in individuals with triglyceride levels below 200 mg/dL. The de Cordova formula showed the most stable performance across varying TG strata, making it a promising candidate for routine use in moderate hypertriglyceridemia. Conversely, the Ahmadi formula exhibited extreme bias and poor clinical utility due to unit incompatibility.

A key limitation of this study is the exclusion of the guideline-endorsed Martin-Hopkins and Sampson-NIH equations, which have shown improved accuracy in low LDL-C and high TG settings. While our retrospective dataset constrained direct application of these methods without reconstruction, we acknowledge that their absence limits the generalizability of our findings to guideline-recommended clinical practice. Consequently, our conclusions regarding the relative utility of Friedewald and de Cordova formulas should be interpreted within this context. Future studies should prioritize the inclusion of these advanced algorithms to ensure alignment with contemporary lipid management standards and improve the clinical relevance of formula-based LDL-C estimation.

## CRediT authorship contribution statement

**Gagua Nino:** Writing – original draft, Validation, Formal analysis, Data curation. **Mokvanidze Lizi:** Validation, Investigation, Formal analysis. **Kekenadze Nino:** Writing – review & editing, Project administration, Methodology, Conceptualization.

## Human ethics and consent to participate

Ethical clearance was secured from the Bioethics International Committee of the Petre Shotadze Tbilisi Medical Academy Academy (identification code: 20230101/01, Tbilisi, Georgia). All procedures adhered to the Helsinki Declaration of 1975, revised in 2013, with participants receiving comprehensive study information and providing oral informed consent.

## Funding

N/A.

## Declaration of competing interest

The authors declare that they have no known competing financial interests or personal relationships that could have appeared to influence the work reported in this paper.

## Data Availability

Data will be made available on request.

## References

[1] Fox K.M., Wang L., Gandra S.R., Quek R.G., Li L., Baser O. (2016). Clinical and economic burden associated with cardiovascular events among patients with hyperlipidemia: a retrospective cohort study. BMC Cardiovasc. Disord..

[2] Derinoz O., Tumer L., Hasanoglu A., Pasaoglu H., Aksakal F.N., Ceyhan M.N. (2007). Cholesterol screening in school children: is family history reliable to choose the ones to screen?. Acta Paediatr..

[3] Silverman M.G., Ference B.A., Im K., Wiviott S.D., Giugliano R.P., Grundy S.M. (2016). Association between lowering LDL-C and cardiovascular risk reduction among different therapeutic interventions: a systematic review and meta-analysis. JAMA.

[4] El Harchaoui K., van der Steeg W.A., Stroes E.S., Kuivenhoven J.A., Otvos J.D., Wareham N.J. (2007). Value of low-density lipoprotein particle number and size as predictors of coronary artery disease in apparently healthy men and women: the EPIC-norfolk prospective population study. J. Am. Coll. Cardiol..

[5] Bruggen FHv, Diamond D.M. (2025). Is targeting LDL-C levels below 70 mg/dL beneficial for cardiovascular and overall health? A critical examination of the evidence. J. Clin. Med..

[6] Burger P.M., Dorresteijn J.A.N., Koudstaal S., Holtrop J., Kastelein J.J.P., Jukema J.W., Ridker P.M., Mosterd A., Visseren F.L.J. (2024). Course of the effects of LDL-Cholesterol reduction on cardiovascular risk over time: a meta- analysis of 60 randomized controlled trials. Atherosclerosis.

[7] Dong J., Guo H., Yang R., Li H., Wang S., Zhang J. (2011). Serum LDL-and HDL-cholesterol determined by ultracentrifugation and HPLC. J. Lipid Res..

[8] Islam S.M.T., Osa-Andrews B., Jones P.M., Muthukumar A.R., Hashim I., Cao J. (2022). Methods of low-density lipoprotein-cholesterol measurement: analytical and clinical applications. EJIFCC.

[9] Martins J., Steyn N., Rossouw H.M., Pillay T.S. (2023). Best practice for LDL-cholesterol: when and how to calculate. J. Clin. Pathol..

[10] Sajja A., Park J., Sathiyakumar V., Varghese B., Pallazola V.A., Marvel F.A. (2021). Comparison of methods to estimate low-density lipoprotein cholesterol in patients with high triglyceride levels. JAMA Netw. Open.

[11] Warade J.P., Dahake H., Kavitha R. (2016). Comparison between direct estimation of LDL and Friedewald's formula. IAIM.

[12] Oliveira M.J.A., van Deventer H.E., Bachmann L.M., Warnick G.R., Nakajima K., Nakamura M. (2013). Evaluation of four different equations for calculating LDL-C with eight different direct HDL-C assays. Clin. Chim. Acta.

[13] Palmer M.K., Barter P.J., Lundman P., Nicholls S.J., Toth P.P., Karlson B.W. (2019). Comparing a novel equation for calculating low-density lipoprotein cholesterol with the friedewald equation: a VOYAGER analysis. Clin. Biochem..

[14] Bairaktari E., Hatzidimou K., Tzallas C., Vini M., Katsaraki A., Tselepis A. (2000). Estimation of LDL cholesterol based on the friedewald formula and on Apo B levels. Clin. Biochem..

[15] Martin S.S., Blaha M.J., Elshazly M.B., Toth P.P., Kwiterovich P.O., Blumenthal R.S. (2013). Comparison of a novel method vs the friedewald equation for estimating low-density lipoprotein cholesterol levels from the standard lipid profile. JAMA.

[16] Martins J., Olorunju S.A., Murray L., Pillay T.S. (2015). Comparison of equations for the calculation of LDL-cholesterol in hospitalized patients. Clin. Chim. Acta.

[17] Sampson M., Ling C., Sun Q., Harb R., Ashmaig M., Warnick R. (2020). A new equation for calculation of low-density lipoprotein cholesterol in patients with normolipidemia and/or hypertriglyceridemia. JAMA Cardiol.

[18] Nigam P.K. (2014). Calculated low density lipoprotein-cholesterol: friedewald's formula versus other modified formulas: calculated LDL-cholesterol. Int. J. Life Sci. Med. Res..

[19] Hattori Y., Suzuki M., Tsushima M., Yoshida M., Tokunaga Y., Wang Y. (1998). Development of approximate formula for LDL-chol, LDL-apo B and LDLchol/LDL-apo B as indices of hyperapobetalipoproteinemia and small dense LDL. Atherosclerosis.

[20] Anandaraja S., Narang R., Godeswar R., Laksmy R., Talwar K.K. (2005). Low-density lipoprotein cholesterol estimation by a new formula in Indian population. Int. J. Cardiol..

[21] Ahmadi S.A., Boroumand M.-A., Gouhari M.K., Tajik P., Dibaj S.-M. (2008). The impact of low serum triglyceride on LDL-cholesterol estimation. Arch. Iran. Med..

[22] Chen Y., Zhang X., Pan B., Jin X., Yao H., Chen B. (2010). A modified formula for calculating low-density lipoprotein cholesterol values. Lipids Health Dis..

[23] Vujovic A., Kotur-Stevuljevic J., Spasic S., Bujisic N., Martinovic J., Vujovic M. (2010). Evaluation of different formulas for LDL-C calculation. Lipids Health Dis..

[24] de Cordova C.M.M., de Cordova M.M. (2013). A new accurate, simple formula for LDL-cholesterol estimation based on directly measured blood lipids from a large cohort. Ann. Clin. Biochem..

[25] Puavilai W., Laoragpongse D. (2004). Is calculated LDL-C by using the new modified friedewald equation better than the standard friedewald equation?. J. Med. Assoc. Thail..

[26] Ramesh J., Selvarajan S., Krishnamurthy S., Sathyamoorthy S.K., Kumar D.S. (2024). Evaluation of 13 formulae for calculated LDL-C using direct homogenous assay in a south Indian population. J. Appl. Lab. Med..

[27] Alsadig R.E.K., Morsi A.N. (2024). Comparison of multiple equations for low-density lipoprotein cholesterol calculation against the direct homogenous method. JLA.

[28] Friedewald W.T., Levy R.I., Fredrickson D.S. (1972). Estimation of the concentration of low-density lipoprotein cholesterol in plasma, without use of the preparative ultracentrifuge. Clin. Chem..

[29] Puavilai W., Laoragpongse D. (2004). Is calculated LDL-C by using the new modified friedewald equation better than the standard friedewald equation?. J. Med. Assoc. Thail..

[30] Mhaimeed O., Burney Z.A., Schott S.L., Kohli P., Marvel F.A., Martin S.S. (2024). The importance of LDL-C lowering in atherosclerotic cardiovascular disease prevention: lower for longer is better. American J. Preventive Cardiol..

[31] National Cholesterol Education Program (1995).

[32] Ephraim R.K.D., Ativi E., Ashie S.A., Abaka-Yawson A., Darkwah K.O. (2023). Assessment of estimated low-density lipoprotein-cholesterol (LDL-c) equations: a systematic review and meta-analysis. Bull. Natl. Res. Cent..

[33] Contois J.H., Warnick G.R., Sniderman A.D. (2011). Reliability of low-density lipoprotein cholesterol, non-high-density lipoprotein cholesterol, and apolipoprotein B measurement. J. Clin. Lipidol.

[34] Hashmi S.B., Ahmed S., Hashmi S., Bux R., Siddiqui I. (2024). Choosing the right equation for calculating indirect LDL-cholesterol (LDL-C) in adult Pakistani population: evaluation of seven equations using big data analytics. Pract. Lab. Med..

[35] Hataysal E.P., Korez M.K., Yesildal F., Isman F.K. (2024). A comparative evaluation of low-density lipoprotein cholesterol estimation: machine learning algorithms versus various equations. Clinica Chemica Acta.

[36] Tomo S., Sankanagoudar S., Sharma P. (2022). Cordova formula for low-density lipoprotein- cholesterol estimation: not only the simplest of all but also superior to other formulae at a higher range of triglyceride. Anatol. J. Cardiol..

