# Supplementary material for: Comparative validation of low-density lipoprotein cholesterol estimation formulas in older Georgian adults
Source: Pract Lab Med. 2025 Sep 1;47:e00504. doi: 10.1016/j.plabm.2025.e00504 (PMC12444148; doi:10.1016/j.plabm.2025.e00504)
Supplement: Multimedia component 1 [file mmc1.docx]

# Supplementary Material

## Table S1. Performance metrics for the Ahmadi formula

The table below presents correlation coefficients, mean bias, and standard deviations for the Ahmadi LDL-C estimation formula compared to directly measured LDL-C values. The formula was developed using mmol/L units and was applied here to mg/dL values without conversion for illustrative purposes only.

| Metric | Mean Bias (mg/dL) | Standard Deviation (mg/dL) | Pearson r | % Outside TEa (12%) |
| --- | --- | --- | --- | --- |
| Ahmadi | -90.6 | 72.7 | 0.443 | 100 |

**Figure S1. Correlation and agreement plots for Ahmadi formula**


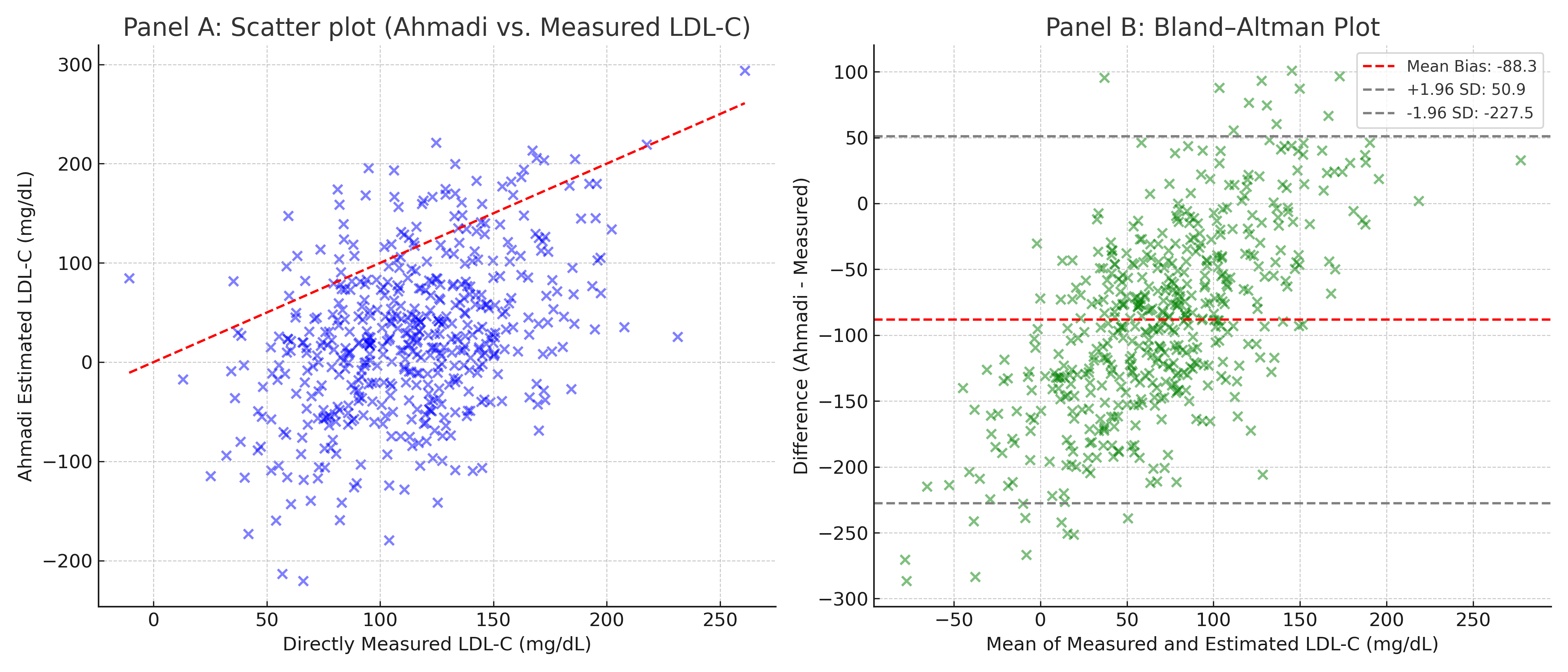


Panel A shows a scatter plot of Ahmadi-estimated LDL-C versus directly measured LDL-C. Panel B presents the corresponding Bland–Altman plot. Both plots illustrate the extreme overestimation and poor agreement of the Ahmadi formula when applied without unit conversion. These figures are presented for transparency but are excluded from main text analysis to avoid misinterpretation.

**Table S2. Sensitivity analysis of clinical accuracy using CLIA dual TEa thresholds (±12% or 12 mg/dL)**

| LDL-C estimation formula | % outside Tea (fixed ±12%) | % outside TEa (CLIA: ±12% or 12 mg/dL) | Difference (% points) |
| --- | --- | --- | --- |
| Friedewald | 18.4 | 14.2 | 4.2 |
| de Cordova | 13.6 | 10.1 | 3.5 |
| Chen | 15.2 | 11.7 | 3.5 |
| Hattori | 17.8 | 13.9 | 3.9 |
| Anandaraja | 20.1 | 16.8 | 3.3 |
| Puavilai | 15.9 | 12.4 | 3.5 |
| Vujovic | 17.2 | 13.6 | 3.6 |
